# An Exploratory Typology of Tobacco-Related Misleading Content on Social Media: Qualitative Analysis of Instagram and TikTok

**DOI:** 10.2196/78854

**Published:** 2025-12-17

**Authors:** Eileen Han, Joanne Chen Lyu, Pamela M Ling

**Affiliations:** 1 Division of General Internal Medicine School of Medicine University of California, San Francisco San Francisco, CA United States; 2 Fondren Library Rice University Houston, TX United States; 3 TSET Health Promotion Research Center, Stephenson Cancer Center University of Oklahoma Health Sciences Center University of Oklahoma Oklahoma City, OK United States; 4 Department of Family and Preventive Medicine, College of Medicine University of Oklahoma Health Sciences Center University of Oklahoma Oklahoma City, OK United States

**Keywords:** misinformation, tobacco control, electronic cigarettes, e-cigarettes, social media

## Abstract

**Background:**

Tobacco-related misinformation on social media platforms presents growing challenges to digital health communication and public health. Although prior studies have focused on platform-specific patterns, a unified framework for categorizing and comparing misinformation across platforms is lacking. Such a framework is essential for improving infodemiological surveillance and designing targeted digital interventions.

**Objective:**

This study was an exploratory analysis aimed to build a cross-platform typology to categorize tobacco-related misinformation.

**Methods:**

Data from Instagram and TikTok between January 2020 and August 2023 were collected using a third-party data collection platform (CrowdTangle) and the TikTok Research application programming interface (API). We reviewed a total of 4850 Instagram posts using a combination of generative artificial intelligence (AI) and human validation by two independent reviewers. In addition, 719 TikTok videos were reviewed manually using qualitative analysis. We iteratively developed and refined the exploratory typology informed by the literature integrating our prior analysis of Twitter data and these new datasets.

**Results:**

Of the 22 (71%) Instagram posts and 9 (29%) TikTok videos we analyzed closely to classify misinformation, 2 (6.5%) were about cigarettes, 22 (71%) were about electronic cigarettes (e-cigarettes), 1 (3.2%) was about heated tobacco products (HTPs), 2 (6.5%) were about nicotine (not mentioning specific products), and 3 (9.7%) were about cannabidiol (CBD) products. 1 (3.2%) post did not mention any type of products. These categories could overlap in a single post. The resulting typology consisted of five core narrative archetypes: false or misleading health claims (A1), wellness and lifestyle appeal (A2), conspiracy-driven policy agenda (A3), undermining trust in science and medicine (A4), and recreational nicotine use normalization (A5). Each archetype has attributes of false claim types and sources. Among the posts we analyzed, A1 and A2 were most likely to be found on Instagram. A3 was most frequently found on Twitter. A4 was commonly seen on both Twitter and TikTok, and A5 was most frequently found on TikTok. Two additional dimensions—type of falsehood and source—were also added to characterize a given misinformation post. This exploratory typology paved the way for a structured lens to view how misinformation is tailored to digital environments and target audiences.

**Conclusions:**

This cross-platform typology building supports digital health research by integrating AI and qualitative methods to categorize tobacco-related misinformation. It can inform the development of automated misinformation detection models, enhance real-time infodemiological monitoring, and guide digital public health campaigns to build tailored countermessaging.

## Introduction

### Background

Misinformation, defined as information that contradicts from the best expert consensus and scientific evidence available at a specific point of time [1], has become a significant focus in health. Although it may or may not have the intention to harm [2], exposure to health misinformation diminishes trust in government institutions and health care providers, reducing compliance with public health guidelines [3,4]. Social media platforms have amplified the reach and speed of misinformation, which tend to be faster and more impactful than fact checking [5,6].

Tobacco-related misinformation refers to information that deviates from established health guidelines or current scientific evidence regarding the relationship between tobacco products and health or does not reflect the true content of tobacco product regulation and policies, posing a unique threat to public health. False or misleading claims about the safety of tobacco products, the risks of nicotine, and the benefits of alternatives (eg, electronic cigarettes [e-cigarettes]) can distort user perceptions, undermine tobacco control efforts, and contribute to disparities in the tobacco-related disease burden [7-9]. Tobacco products and their marketing are rapidly evolving [10]. Many related issues about tobacco products, especially the new products that deliver nicotine (eg, e-cigarettes, nicotine pouches) and those offering a noncombustible way of consuming tobacco (eg, heated tobacco products [HTPs], smokeless tobacco), have not been resolved, with ongoing controversies and debates, which makes the boundaries of tobacco-related misinformation fluid and requires frequent updates to remain relevant [1]. It is essential to gain a deeper understanding of the types and common characteristics of misinformation on major social media platforms to facilitate targeted health information literacy education for health interventions.

Despite increasing concern about tobacco misinformation online, research in this area remains fragmented. Previous studies have identified thematic categories, such as misinformation about nicotine addictiveness or vaping risks [8], but lack consistent criteria for categorizing misinformation across platforms. Most work to date has primarily focused on one platform, Twitter (now X), and has mostly discussed user experiences, thematic analysis of health effects, and promotional strategies [11], with limited attention to how different kinds of misinformation can be sustained by different platforms. Prior studies have developed taxonomies to classify cannabidiol (CBD) products and electronic juices (e-juices) based on content characteristics, demonstrating the utility of structured frameworks for analyzing related products [12,13]. However, attempts to structurally categorize tobacco-related misinformation—particularly on social media—remain limited.

### Aim of the Study

To address these gaps, we built an exploratory typology of tobacco-related misinformation. Our typology was based on the established health misinformation taxonomies that mainly came from the domain of vaccines [14-16]. We adapted key elements and structures to fit the tobacco context. Specifically, we built on the use of thematic categorization at multiple levels (eg, themes and concerns, adverse effects, freedom, and distrust in science) [14-16], while also incorporating other dimensions, such as source types [15]. We further extended these frameworks by making different falsehood types as a unique dimension, which were implied in the existing frameworks (eg, conspiracy) [16], to better capture how misinformation manifests at different message strategies and actor motivations. The typology presented in this study was also drawn from a preliminary framework of tobacco-related misinformation based on Twitter data, which included content (cessation, health effects, policy, and substances), types of falsehood (unsubstantiated, misrepresentation, conspiracy), and sources (advocacy group, individual advocate, influencer/individual, and retail) [17].

Expanding on our analysis of Twitter [17], we analyzed data from Instagram and TikTok to modify the framework. We introduced five archetypes at the core of this typology, expanding the content themes to include key thematic issues and corresponding persuasive strategies commonly used to spread misinformation about tobacco products, together with two other dimensions—sources and false claims. Our goal was to inform future efforts to identify, classify, and monitor tobacco-related misinformation across platforms and to support the development of tailored digital public health interventions.

## Methods

### Data Collection

Social media posts were collected from Instagram and TikTok. Instagram data from January 1, 2020, to May 31, 2023, were obtained from CrowdTangle, a third-party social media data collection platform run by Meta (closed since August 2024). The search keywords were informed by our previous Twitter study [17] and were derived from those used to search a large database collecting tweets related to tobacco since 2015 [18], resulting in 385,312 posts in English (Multimedia Appendix 1).

TikTok videos were collected using TikTok’s Research application programming interface (API). Due to the restrictions at the time of data collection, only videos that were public and created by users in the United States aged 18 years and older were available. Based on the analysis of Twitter [17] and Instagram data, we found that popular hashtags were often clustered together. For example, “vape”-related hashtags were likely to appear in the same post, so only central key terms such as “vape,” “vaping,” “e-cig,” “ecig,” “tobacco,” and “nicotine” were used as our search terms (Multimedia Appendix 1), and the first 100 videos (or if <100, the number of videos on the first page) resulted from the API search of each month were collected to maintain a sample size suitable for human coding. Next, among the collected videos, 10% were randomly selected for further analysis, proportional to the number of videos collected for each month from January 1, 2020, to August 31, 2023. In total, 719 videos were sampled for further analysis.

Our unit of analysis was a single post (on both platforms). A “post” was defined as content with a unique identifier in the dataset (usually a unique post ID) that has information such as a username and media content (text, single or multiple images, video, audio, animation), together with comments under the same unique identifier. In this study, we mainly focused on the text associated with each Instagram post, and for TikTok, our focus was the transcribed audio in each video we analyzed.

We developed a set of selection criteria based on the literature [7,8] and guidelines from established health authorities to determine whether a given post contained misinformation. We used the Centers for Disease Control and Prevention (CDC) and World Health Organization (WHO) guidelines because they provide foundational guidance for public health practices in the United States and global contexts, respectively.

Our “ground truth” included the following [19-22]:

- Cigarette smoking is the leading cause of preventable disease in the United States, harms nearly every organ [19], and kills more than 7 million people worldwide each year [20]. Evidence-based cessation programs have proven effective and (counseling and medication) are the recommended best practice [19,20].
- There has is no evidence suggesting that HTPs reduce health risks or could help with smoking cessation.
- E-cigarettes are relatively safer than cigarettes when adult users completely switch to this product. Although there is evidence of efficacy in cessation in randomized trials, there is no evidence of e-cigarettes being an effective cessation tool when used as consumer products at the population level [21].
- People who never smoked/used e-cigarettes or HTPs should not start. Children and adolescents should not use these products [21,22].

Additionally, we prompted ChatGPT to produce a list of tobacco misinformation selection criteria and applied these criteria to the selection of misinformation posts. ChatGPT is an artificial intelligence (AI) system developed by OpenAI that uses a large language model (LLM) to generate human-like text responses based on user input. LLMs belong to a broader class of technologies known as generative AI, which produces new content, including text, images, and audio, by learning patterns from extensively large amounts of text datasets used to train the models [23].

The ChatGPT-produced criteria were as follows:

- Claim that vaping or e-cigarette use is completely safe or significantly safer than traditional tobacco use without acknowledging the existing health risks.
- Promote tobacco products as a healthy alternative to smoking.
- Suggest that tobacco products have health benefits.
- Target younger audiences or nonsmokers, encouraging them to start using tobacco products.
- Misrepresent the addictive nature of nicotine or tobacco.

### Data Processing

**Figure 1:**
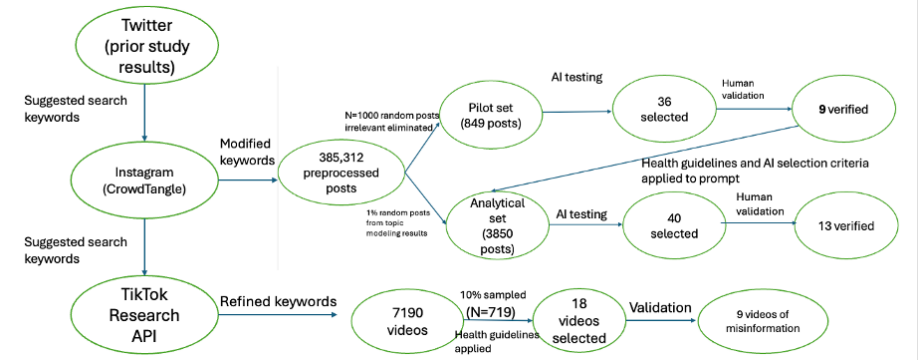
Multistep data processing and analysis of Instagram and TikTok posts with AI assistance (the analysis of Twitter data has been presented in a separate manuscript [17]). AI: artificial intelligence; API: application programming interface.

Sampling involved multiple steps (Figure 1). Due to the large volume of posts collected, a small subset of data was selected for analysis using a combination of computational and manual techniques. Our typology was based on a close reading of a small number of posts that met conservative criteria for classification as misinformation.

For Instagram data, we performed an initial investigation of top users (number of followers and number of posts). We filtered out likely irrelevant content using a combination of keywords and accounts based on our initial investigation (eg, accounts that marketed sportswear). For the remaining posts, we applied Latent Dirichlet Allocation (LDA) topic modeling on all the preprocessed texts and selected a four-topic solution with the highest coherence score. Although not perfect, these measures helped us gauge the general patterns of what had been discussed in the collected posts.

A pilot set of 1000 randomly sampled posts was randomly selected. Generative AI was then used to identify potential misinformation, and 36 posts (3.6%) were identified. Human validation of the 36 posts retained 9 (25% precision rate) of those. We did not re-examine the entire sample.

Based on this pilot test and validation, 1% of the posts proportional to the size of each topic were screened using AI (N=3850). ChatGPT identified 40 posts, and 13 (32.5% precision rate) of them were validated by human reviewers and included in the final set (total n=22).

TikTok videos could not be processed using AI tools at the time of the study due to technical limitations. Instead, a 10% random sample (n=719) was manually reviewed and coded by researchers using predefined criteria. The lead author performed the initial screening of the 719 videos for relevancy and potential misinformation candidates. The candidate videos (n=18, 2.5%) were coded by two other researchers one after another: an expert with decades of experience in tobacco control research and a research assistant who is a TikTok user and familiar with the platform. For the initial review, the three reviewers agreed on 10 (55.6%) of 18 videos as containing misinformation. After the three rounds of review and a subsequent group discussion, we reached a consensus on the final set of posts that met the criteria for misinformation (n=9, 90%). Additional notes were also taken to record the positive or negative portrayals of vaping (pro- or antivaping) in the posts, and we coded a few content themes, such as vaping tricks, product promotion, customization, education [24], and health. The ground-truth criteria were applied to each video examined.

To identify misinformation, we compared each post AI identified with the ground truths and looked for claims (or components in the claims) that were inconsistent with the ground truths. The following were categorized as misinformation:

- Any claims that downplayed the harm of cigarettes [19,20]
- Any claims that e-cigarettes or HTPs can be used for smoking cessation at the population level or implying that e-cigarettes are effective cessation devices for everyone [21]
- Any claims encouraging people to use these products or advocating accessibility to these products regardless of age or tobacco use history
- Any claims that suggested any of these products have health benefits without restrictions (age, use history)
- Any posts that claimed their products are “FDA approved” or are “pending approval by FDA” (where “FDA” refers to the Food and Drug Administration) or any posts claiming their products have therapeutic functions [25]

### Data Analysis

Applying the previously developed analytical framework based on the analysis of selected tweets with misinformation [17], we coded the 31 selected posts and videos (n=22, 71%, posts from Instagram and n=9, 29%, videos from TikTok) according to their major claims, types of falseness, and sources. Two researchers discussed the categories they developed and deliberated on possible modifications of the categories, including merging, splitting, regrouping, and expanding them, as well as refining the definitions and renaming the categories. This was done with reference to other existing misinformation taxonomies [14,15].

Table 1 shows a modified version of the misinformation framework we developed on the basis of Twitter data analysis in a prior study [17]. The “targeted issue” dimension used to be “content,” which included four major themes based on the results from Twitter data analysis [17]. In this modified framework, the themes “cessation aid,” “health effects,” and “substances” were further consolidated into a “health” category, making the targeted issue either health or policy related, as we noticed that most of the posts we analyzed contained a combination of the three major themes. We also modified the source dimension by combining vaping advocacy groups and individual vaping advocates to “vaping advocacy (group and individual),” divided “unspecified individuals/influencers” into two categories, and expanded the “retail stores” category to include “business entities” to further include accounts of brands and online stores.

**Table 1:** Misinformation framework.

Dimension and subcategory			Definition		Example (redacted)
**Targeted issue [26]:** **the general issue or broader topic that the misinformation is focused on**					
	Health (cessation, health effects, wellness, substances)	Claims about a product that has health benefits, can be a safe alternative to cigarettes, or can be effective in smoking cessation or claims about the addictiveness or benefits of any substances		“It’s not only a tool to help you quit smoking, but also has health benefits for all users…” (Instagram)	
	Policy	Claims about policies and regulations regarding any tobacco products		“Come out as a vaper! There’s no shame in drinking coffee, so why be ashamed of using safer nicotine? The more people know someone who quit smoking through vaping, the more we can gain support for harm reduction!” (Instagram)	
**Falseness of claim:** **how claims deviate from the ground truths by different ways evidence was used**					
	Misrepresentation	A partial presentation of evidence to support a claim, including overstatement (exaggeration or false comparison), out-of-context use of data/scientific evidence, selective use of scientific evidence (cherry-picking), or using anecdotal evidence for generalization		“If you’re addicted to nicotine like billions are to caffeine, which would you choose—smoking or vaping? Both nicotine and caffeine are plant-based alkaloids, affect the brain similarly, and are equally addictive.” (Instagram)	
	Distortion	A distorted use of evidence, often appearing to be conspiracy narratives on powerful entities taking away individual freedom and interests		“When all vape devices are banned, I hope we’re not weak enough to go back to big tobacco like the government wants us to.” (Twitter)	
	Unsubstantiated claim	Claims that have no existing scientific evidence to support as of now or use made-up evidence to support them		“Stay focused with our new energy drink flavor. Available now. #newflavor #disposable #vape #rechargeable” (Instagram)	
**Source:** **categories of the authors of the posts**					
	Vaping advocacy (group and individual)	Known advocacy groups or individuals for tobacco harm reduction or policies in favor of industry interests		“Come out as a vaper! Don’t hide your use of safer nicotine. Normalize it like coffee!” (Instagram)	
	Influencer^a^	Individuals who have claimed certain areas of expertise and market products, with paid or unpaid promotions of certain products		“Not just for quitting smoking—our new device offers wellness benefits too. Check out the latest model from our store!” (Instagram)	
	Individual	Organic users who did not claim to be affiliated with any known organizations or companies that sell products or advocate provaping policies (regardless of whether they are, in fact, affiliated)		“I just realized how harmful vaping really is. People don’t know what it does to you—it causes depression and anxiety!” (Twitter)	
	Business entity (retail store, brand, company)	Online accounts of stores or brands		“Reminder: This product may contain nicotine. Nicotine is only mildly addictive and not carcinogenic.” (Instagram)	

^a^Since we could not verify the real identity of a user but could only infer from the account information that is publicly available and collected in the dataset, we defined an influencer as someone having posted consistently on sponsored content (marked as “sponsored” in both Instagram and TikTok), promoting a product (having a “link in the bio” to redirect users to purchase or join an event), or claiming to have some expertise in a knowledge domain.

Next, we expanded the targeted issue dimension into a set of archetypes, as we found that the organizing logic of misinformation is often a combination of themes and persuasive intentions. Reviewing the types of content themes revealed that “policy” is more of a persuasive goal, in addition to being a theme, because the health themes were often found in posts that advocated for more relaxed regulation of products like e-cigarettes. We also found new themes that we did not capture in the previous analysis of Twitter, that is, the theme about wellness associated with certain products. In addition, the analysis of TikTok videos revealed another type of message with elements of entertainment that the previous study of Twitter did not capture. Therefore, we reconsidered “policy” as an element of persuasive goals, and then, all together, we identified three persuasive goals from reviewing the targeted issues presented in the posts: marketing, policy advocacy, and entertainment and socialization. We then categorized the posts into five misinformation archetypes based on how they intersected with these thematic and persuasive elements. The falseness of claims and the sources disseminating them functioned as descriptive attributes.

### Ethical Considerations

The data of this study came from publicly available social media data. The analysis was solely observational and did not involve human subjects. When presented, the posts were anonymized, and other information that could make the users identifiable was removed. When quoted as examples, the original text in each post was redacted.

## Results

### Tobacco Products Featured

Of the 31 posts and videos we analyzed (n=22, 71%, posts on Instagram and n=9, 29%, videos on TikTok), 2 (6.5%) were about cigarettes, 22 (71%) were about e-cigarettes, 1 (3.2%) was about HTPs, 2 (6.5%) were about nicotine (not mentioning specific products), and 3 (9.7%) were about CBD products.

### Five Archetypes of Tobacco Misinformation

We identified five archetypes, as listed in Table 2: false or misleading health claims (A1), wellness and lifestyle appeal (A2), conspiracy-driven policy agenda (A3), undermining trust in science and medicine (A4), and recreational nicotine use normalization (A5). The five archetypes were derived from the content themes of misinformation posts and grouped according to their primary persuasive intent—marketing and promotion, policy advocacy, or entertainment and socialization—to show the common content strategy certain misinformation archetypes share but may express with different message characteristics. We listed the definition of each archetype and examples found in our sample (Table 2).

**Table 2:** Archetypes and tobacco-related misinformation^a^.

Archetype; posts and videos (N=31), n (%)	Definition	Attributes	Persuasive goal	Platform	Example (redacted)
A1: false or misleading health claims; 15 (48.4)	Promoting products with unverified, misleading, or deceptive health claims	Unsubstantiated/misrepresentation Business entity/influencer	Marketing and promotion	Instagram	“It’s not only a tool to help you quit smoking, but also has health benefits for all users…” (Instagram)
A2: wellness and lifestyle appeal; 5 (16.1)	Promoting products as compatible with a healthy, active, or wellness-oriented lifestyle	Unsubstantiated/misrepresentation Business entity/influencer	Marketing and promotion	Instagram	“Stay focused with our new energy drink flavor. Available now. #newflavor #disposable #vape #rechargeable” (Instagram)
A3: conspiracy-driven policy agenda; 3 (9.7)	Advocating for relaxation of regulations on e-cigarettes^b^ and other new products through conspiracy narratives	Misrepresentation/distortion Advocacy	Policy advocacy	Twitter	“When all vape devices are banned, I hope we’re not weak enough to go back to big tobacco like the government wants us to.” (Twitter)
A4: undermining trust in science and medicine; 5 (16.1)	Casting doubt on health authorities, scientific research, or medical professionals to undermine public trust to push for relaxation of regulations	Unsubstantiated/misrepresentation Advocacy/influencer	Policy advocacy	TikTok, Twitter	“Diacetyl causes what’s known as ‘popcorn lung.’ But that’s not even present in vape liquids anymore. So where’s your research?” (TikTok)
A5: recreational nicotine use normalization; 3 (9.7)	Using entertainment elements to downplay product risks and normalize use through engaging, shareable content	Unsubstantiated/misrepresentation Individual/influencer	Entertainment and socialization	TikTok	A video with National Basketball Association (NBA) footage with the caption that a vaper’s lung function is just as good as that of the athletes (TikTok)

^a^A post may belong to one or more archetypes at the same time.

^b^e-cigarette: electronic cigarette.

**Figure 2:**
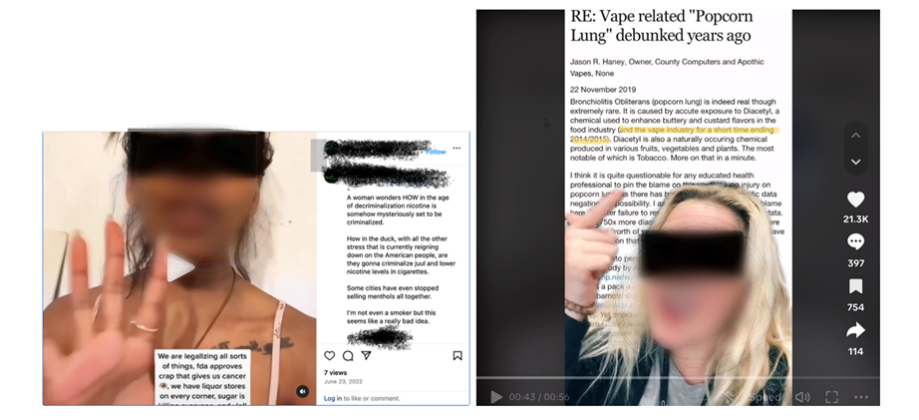
Screenshots of two examples in our analyzed posts that contain misinformation about vaping policy and health-related issues.

“False or misleading health claims” (A1) typically involve marketing-oriented messages that promote products (especially e-cigarettes) using unverified or deceptive claims about cessation benefits or reduced harm. “Wellness and lifestyle appeal” (A2) similarly reflect marketing goals but emphasize compatibility with fitness, wellness, or clean living, often downplaying health risks by associating products with aspirational lifestyles. “Conspiracy-driven policy agenda” (A3) represents advocacy-driven content that opposes regulation by framing public health policy as driven by hidden motives or corporate collusion. “Undermining trust in science and medicine” (A4) also serves an advocacy function but focuses on eroding public confidence in scientific evidence or health authorities, often by highlighting inconsistencies or perceived bias in medical guidance. Finally, “recreational nicotine use normalization” (A5) leverages entertainment and socialization strategies, such as humor, memes, or influencer-driven trends, to trivialize potential harms and portray tobacco product use as socially acceptable, particularly among youth (Figure 2).

For each archetype, typical attributes from the “falseness of claim” and “source” dimensions were added.

#### Marketing and Promotion (A1 and A2)

“False or misleading health claims” (A1) and “wellness and lifestyle appeal” (A2) primarily serve promotional and marketing purposes, aiming to sell products by leveraging health-related and wellness-oriented messaging. Of 22 (71%) sample Instagram posts containing misinformation, 21 (95.5%) focused on health-related content. Promotions frequently adopt misleading descriptors such as “natural,” “safe,” “slightly addictive,” “just like caffeine,, and “no smell” to downplay risks and use inaccurate or unsupported descriptions of product features and health effects, such as a product having “health care features,” helping people “stay focused.” They also make sweeping claims that a product is “a better choice in every sense” regardless of tobacco use history, health condition, or age.

Examples of these archetypes were most frequently observed on Instagram and were typically characterized by unsubstantiated claims or misrepresentations. The sources were predominantly business entities or influencers. Common examples included claims that nicotine products are natural, therapeutic, or safer alternatives to smoking and assertions that cannabis or vaping could aid cessation or improve wellness.

Of the 22 (71%) posts, 10 (45.5%) featured unsubstantiated claims of health benefits, while the remaining included misrepresentations, such as minimizing nicotine’s addictiveness (“nicotine is just like caffeine”) or making universal safety claims. Business entities were the most frequent source (n=14, 63.6%), including retail stores and official accounts of brands, followed by influencers (n=5, 22.7%).

#### Advocacy (A3 and A4)

“Conspiracy-driven policy agenda” (A3) and “undermining trust in science and medicine” (A4) fall under advocacy, as they seek to influence public opinion and regulatory policy. According to our prior data and findings [17], examples of these archetypes were mostly found on Twitter and TikTok and were typically from advocacy groups or individuals. The claims within these archetypes often involved misrepresentation or distortion. A3 was mostly found on Twitter [17], with the typical argument that the government is trying to ban vape to help Big Tobacco kill more people by not allowing people to access the “healthier” alternative that could help them quit smoking. One TikTok video also contained the conspiracy theory that the outbreak of EVALI in late 2019 was COVID-19 covered up by the government.

Posts belonging to A3 and A4 were most likely seen on Twitter in our prior study [17]. In our TikTok data, 6 (66.7%) of the 9 videos were categorized as A4, with typical claims that discredited research showing the harms of vaping, arguing that e-cigarettes should be accessible to all for their health benefits.

#### Entertainment and Socialization (A5)

“Recreational nicotine use normalization” (A5) aims at entertainment and socialization, and of our 9 (29%) identified TikTok videos, 3 (33.3%) belonged to this archetype. This archetype captures health-related content that normalizes tobacco product use—especially vaping—by embedding it in casual, entertaining, or socially relatable contexts. The goal is often implicit normalization through humor, storytelling, dramatization, or informal advice and typically lacks explicit political or direct marketing agendas. These posts may involve music, memes, or skits and can also include peer-to-peer communication, such as personal anecdotes or tips framed as everyday wisdom (eg, “I was vaping while pregnant, and now my baby is fine. It’s completely fine to do that.”). These posts primarily contained unsubstantiated health claims and were posted by individuals, many of whom may not overtly identify as advocates or marketers but still contribute to the normalization of risky behaviors.

## Discussion

### Principal Findings

This study developed an exploratory typology of tobacco-related misinformation, centralized on five distinct archetypes—false or misleading health claims, wellness and lifestyle appeal, conspiracy-driven policy agenda, undermining trust in science and medicine, and recreational nicotine use normalization—and their attributes of falseness of claims and source types, circulating on Twitter, Instagram, and TikTok between January 2020 and August 2023. This study extended prior work on health misinformation taxonomies [14,15] and the tobacco misinformation framework [17] by integrating a hierarchical [15,27], multidimensional classification approach that differentiates misinformation by content and intention, as well as how misinformation is created, manipulated, and disseminated.

This classification provides a structured lens for understanding how misinformation is tailored to different platforms and potential audiences, helping gain insights into the complexity of tobacco-related misinformation by identifying common patterns in message framing, the actors involved, and their strategic objectives. These insights contribute to further developing a more structured taxonomy for automated detection models, targeted interventions, and misinformation mitigation efforts.

#### Multidimensional Framework for Classifying Tobacco Misinformation

In this study, we intended to explore how tobacco-related misinformation instances can be classified across platforms. Although the typology resulted from a small number of cases, the classification is based on ground truths and resonates with the practices of the tobacco industry over time and the common themes of social media discussion about tobacco-related products.

The archetypes center on tobacco-specific themes that form a stable foundation for categorizing misinformation. These archetypes are grounded in topics consistently identified in the prior literature on tobacco product marketing and misinformation [8,28], reflecting established thematic patterns rather than transient or emerging topics. Given the relative stability of these tobacco-specific themes, it allows for a clear differentiation of misinformation based on its primary themes and persuasive goals, providing a solid basis upon which to explore the other dimensions—“falseness of claim” and “source”.

The archetypes were developed based on clear thematic groupings. Although most of the posts contain issues about health, the ways health-related claims are used to address policy or to entertain and share personal advice are different from using health claims to sell products. Therefore, the archetypes are grouped to reflect their primary persuasive goal.

A1 (false or misleading health claims) consolidates health-related subthemes, acknowledging that many Instagram and TikTok posts in our sample address multiple health topics, including vaping safety, cessation, and the addictiveness of nicotine. A2 (wellness and lifestyle appeal) captures content that promotes tobacco products using wellness-related terms (eg, “energy boosting”) that are not exactly about health effects. A1 and A2 are mostly marketing posts. These archetypes highlight how commercial actors exploit gaps in consumer knowledge and regulatory oversight, ultimately aiming at normalizing harmful products. This deceptive marketing strategy has long been used in the tobacco industry, where companies leverage health-related descriptors such as “plant based,” “light,” or “natural” to create a misleading perception of reduced harm in tobacco products [29-32]. Another critical component of marketing misinformation is the misrepresentation of regulatory status. Although not found in our sample (were seen in posts not included in the sample), claims such as being “FDA approved” can mislead consumers into assuming that these products have been scientifically validated as safe [25,33].

Policy-related misinformation topics were categorized into two distinct archetypes (A3 and A4), both of which advocate for a relaxed regulation of products like e-cigarettes, rooted in distrust toward established authorities [7,18]. However, they present distinct narratives: although A3 uses conspiracy rhetoric to discredit government tobacco control policies, A4 advances antiscience narratives that dismiss expert consensus on health risks. Together they demonstrate how advocacy groups and individuals could influence public opinion and undermine regulatory efforts, often by framing restrictions as corporate overreach or an infringement on personal freedoms. Industry-aligned groups have been pushing for more relaxed e-cigarette regulation for their commercial interests [34], and their activities could play a major role in shaping consumer perceptions into believing that restricting e-cigarettes could lead to smoking relapse and essentially benefit Big Tobacco [35].

Lastly, A5 (recreational nicotine use normalization) pertains to content that normalizes tobacco product use—primarily vaping—through humor, drama, music, and engaging content to achieve popularity and virality [36]. Posts classified in this archetype positively portray vaping and make the use of tobacco products appear fun, not only socially acceptable but also woven into various lifestyle domains, potentially influencing audience perceptions and behaviors [36,37]. The TikTok algorithm also amplifies vaping content with entertainment features despite the platform’s intentional removal of vaping-related hashtags [38]. Historically, tobacco companies have integrated their branding into entertainment-oriented events, such as video games, sports, music concerts, and associate smoking with youth culture, rebellion, and social status to sell their products [39-41]. These tactics are particularly effective in targeting young audiences, exposing them to misleading content, increasing the likelihood of their interaction with the promotional materials and the initiation of use [42,43].

The “falseness of claim” dimension complements the archetypes by offering a granular breakdown of how misinformation is constructed or manipulated. It allows researchers to infer (although not to confirm) whether a claim likely stems from a genuine misunderstanding or intentional deception. The three subcategories reflected variations in evidence support and manipulation. *Unsubstantiated* claims lack supporting evidence [44,45], while *inaccurate* descriptions are the characteristics of *misrepresentation* of true or partially true information through selective presentation or misunderstanding [45,46]. *Distortion* involves deliberate manipulation, often aligning with broader conspiracy narratives [44], such as the collusion between Big Tobacco and the government, mirroring patterns found in common medical conspiracy theories [47], and extending across various health domains [26,48].

The source dimension further enriches the typology by identifying actors disseminating misinformation and their potential affiliations, which also gives researchers clues for the possible intentions in each post. We distinguished between individual users and those with ties to interest groups, including business entities, advocacy, and influencers. Our findings highlight the prominent role of vaping advocates in spreading misinformation, primarily on Twitter [17] and TikTok [24], and their centrality in dissemination networks [49]. Within the advocacy category, we differentiated between individuals and organized groups to assess whether activities may be coordinated. Although beyond the scope of this research, studies have shown that advocacy groups may have industry links and play a role in shaping public perceptions and opposing tobacco control efforts [50]. Influencers promoting vaping products also emerged as a significant source, although many did not claim expertise in vaping, often promoting other products, such as cosmetics or supplements. Retailers and brands were primarily found on Instagram [51-53] and are now named “business entities” as the new source category to emphasize their commercial intent.

Together, the core archetypes and two supporting attribute dimensions (“falseness of claim” and “source”) provide a structured and scalable framework for categorizing tobacco-related misinformation in digital environments. By centering on archetypes rooted in well-established tobacco-related themes, the exploratory typology offers a stable lens to track dominant misinformation narratives across platforms. The attribute dimensions enrich this framework by highlighting variations in message veracity and identifying the actors who propagate these narratives. This layered structure supports more precise detection and interpretation of misinformation, paving the way for a more systematic classification that will enable digital health researchers and practitioners to align countermessaging strategies with both content type and source characteristics.

Tobacco misinformation is not monolithic; rather, it is highly contextual and strategically adapted to resonate with different audiences and platform specifics. By mapping how false claims and sources intersect with narrative archetypes, this framework enables the design of nuanced, audience-centered interventions by tailoring messages to challenge specific categories.

#### Challenges in Identifying and Categorizing Tobacco-Related Misinformation

Identifying tobacco-related misinformation is a challenge, given the variability in the broader definition of health misinformation [46] and the implicit nature of many misleading claims. Although unsubstantiated claims about the health benefits of tobacco products are easily detectable, much of the misinformation present in this study is subtle. Implicit misinformation, such as the misuse of true claims, may be perceived as less false than fabricated claims, posing additional challenges for correction efforts [45].

For example, in some posts, the description of nicotine as “slightly addictive” within lengthy product descriptions at retail store accounts is put alongside other true statements in the posts. Similarly, cessation claims for vaping products, although potentially true at an individual level, become misleading when used for marketing, as population-level evidence is lacking. Some of the falseness of claims needs readers to be meticulous about the details; for example, although HTPs, such as IQOS, have been authorized by the FDA as a “modified risk product,” it does not mean complete safety to use HTPs regardless of one’s age, smoking history, or existing health conditions, and a sweeping claim that HTPs are “a better choice for everyone” would be a misrepresentation of the truth. Other variability is related to how AI was used in assisting the identification. It mistakenly identified posts with keywords such as “safe,” even though the posts themselves were about relative safety.

TikTok videos further complicate detection, as humor and entertainment elements often obscure the seriousness of the content [36]. For example, in the video about smoking and elevated testosterone levels, comical, exaggerated, and unrealistic visual elements are used to illustrate the claim, which is a misrepresentation of scientific research to argue for smoking and hormonal and fitness benefits, to normalize and even glamorize cigarette smoking.

In both datasets, particularly Instagram, we found a notable number of posts related to CBD products. Although we did not include CBD-related terms in our search string, the appearance of CBD content was largely due to the inclusion of terms related to vaping and vaping devices. Although CBD was not the focus of this study, posts about CBD could still fit in some archetypes, particularly “false or misleading health claims” and “wellness and lifestyle appeal.” It shows that our classification system is generalizable to other substances. Given the rising popularity of CBD products, the presence of these posts in our final analysis is significant as it shows that the boundaries of products can be fluid, and the platform algorithm could feed users content with misleading claims of other products based on their browsing history. Future research or interventions about tobacco-related content should also consider the impact of this possible content spillover.

Although the number of posts and videos identified as containing misinformation was relatively small, this reflects the highly selective nature of our analytical approach rather than the overall prevalence of misinformation in the broader dataset. For Instagram, only a subset of the full dataset (approximately 1%) was screened using a generative AI model informed by topic modeling outputs, and only those posts flagged by AI were subject to human validation, which means that some posts containing misinformation might have been missed if AI did not successfully pick them up. This conservative, multistep process prioritized precision over recall, meaning that additional misinformation examples may have been missed due to reliance on AI’s initial filtering. For TikTok, a 10% random sample was manually reviewed by researchers without AI assistance due to technical limitations at the time of data collection. As such, the limited number of identified misinformation posts should not be interpreted as a measure of prevalence but rather as a curated set for developing and validating a future taxonomy.

Although this study presented a limited set of qualitatively analyzed posts, it was designed to allow for iterative refinement and structured updates as new misinformation instances are observed across platforms. New instances would be flagged when they introduce novel categories in one or more of the three dimensions (eg, AI-generated sources, tobacco industry posts) or their subcategories (eg, politicians or celebrities endorsing a product) or introduce new families of products. These could be identified through ongoing social media monitoring, thematic analysis of more data, and input from community partners. Proposed updates should be evaluated to see whether they fit within the existing structure or suit for new categories or subtypes.

### Future Directions

There are significant gaps in understanding the effects of tobacco-related misinformation on knowledge, attitudes, and behaviors, feelings, norms, and trust [54,55] in tobacco-specific messages. Long-term intervention strategies should prioritize health information literacy [56], equipping audiences with critical skills to assess source credibility, recognize hidden intentions (eg, marketing and advocacy), and identify common misinformation tactics. Tobacco control efforts in public health agencies need to implement real-time monitoring and adaptive responses to identify and address emerging misinformation trends using the taxonomy to identify problematic messages.

Future work should focus on expanding the exploratory typology using a larger sample of posts to inform development of a taxonomy. Specific directions and steps include (1) conducting a systematic analysis with larger sample sizes to build a taxonomy, (2) validating the taxonomy’s reliability and generalizability across different datasets and platforms (eg, Facebook, Reddit, YouTube) and by AI and human researchers, and (3) incorporating the taxonomy into automated detection tools for real-time monitoring.

### Limitations

This study has limitations. The data collection methods were different across platforms due to the different tools available at the time of research for the three platforms. The keywords used for the search were leaning toward e-cigarettes or HTPs, not including smokeless tobacco and oral nicotine products, so the findings may not be fully generalizable across product types, suggesting future expansion when developing into a taxonomy.

The small subset of the collected data for analysis and the focus on precision in human validation of AI results significantly limited the robustness of misinformation classification, but the archetypes we identified are closely aligned with the major findings in the literature about misleading contents of tobacco-related products, indicating that this classification system can be applicable to larger datasets, which we will test in our next steps.

At this exploratory stage, our focus was not fine-tuning the AI model to reach a higher consensus with human researchers. More sophisticated prompting, iterative interactions, and different model selections could improve the performance of AI assistance, making it better serve the purpose of automating the tasks of finding misinformation content in large volumes of texts.

Some of the subcategories need more specific measures. For example, for “misrepresentation,” the definition is broad and includes anything that is not an accurate representation of a scientific finding. However, it may not be operationalizable to determine whether a claim is a misrepresentation by an exhaustive search of all the existing literature. A clear threshold is needed; for example, if a certain number of references from credible sources support a claim, it should be considered as true. Although the archetypes provide a well-established set of themes in tobacco-related misinformation, as new products emerge, it is necessary to periodically revise the taxonomy to catch up.

The analysis was primarily based on texts; both the transcribed texts from the audio of the TikTok videos and captions associated with Instagram posts, and thus the visual cues from these two platforms, were largely missing. Although we did consider the visual elements when analyzing the posts, these visual cues themselves were only serving an auxiliary role in determining whether a post met our misinformation criteria. Such elements could be the youth-appealing appearance of the influencer, the perceived young age of a model, or the use of popular cultural symbols. Since our focus was on the inaccurate message, we did not analyze whether such visual elements, although not containing such a message, could have misleading effects. Therefore, the visual elements were not considered as a separate archetype or a dimension in this classification attempt. The missing of visual elements could be addressed in the next step of this research, which involves user reactions to these posts, and additional insights could be gained into how misinformation was perceived.

Finally, in our datasets, the tobacco industry presence is low. This is largely due to restrictions of data collection and platform policy. We were only able to collect publicly available data, while major tobacco companies make their US accounts private or set age restrictions. None of the identified posts came directly from the industry, but some of them were marketing posts, illustrating the marketing influence of the industry. Future expansion of our typology could include the tobacco industry as a source of misinformation.

### Conclusion

The exploratory typology developed in this study provides a foundational framework for systematically identifying and addressing tobacco-related misinformation across social media platforms, preparing for the development of a structured taxonomy for scalable infodemiological monitoring and intervention planning. By integrating computational and qualitative analysis, this framework captures stable, theme-based archetypes, alongside critical attributes, such as source type and claim falseness. This approach supports the development of automated detection tools, including machine learning models to recognize narrative patterns, misinformation tactics, and key misinformation actors. It also prepares for the design of targeted digital health interventions by enabling public health practitioners to tailor countermessaging strategies to specific archetypes, source types, and audience vulnerabilities.

## Data Availability

The datasets generated and analyzed during this study are not publicly available due to the inclusion of personal identifiable information in the posts we collected. Deidentified and redacted versions will be available from the corresponding author upon reasonable request.

## Appendix

Multimedia Appendix 1 Search keywords.

## Abbreviations

AI: artificial intelligence
API: application programming interface
CBD: cannabidiol
e-cigarette: electronic cigarette
FDA: Food and Drug Administration
HTP: heated tobacco product
LLM: large language model
