# Supplementary material for: An Exploratory Typology of Tobacco-Related Misleading Content on Social Media: Qualitative Analysis of Instagram and TikTok
Source: J Med Internet Res. 2025 Dec 17;27:e78854. doi: 10.2196/78854 (PMC12756659; doi:10.2196/78854)
Supplement: Multimedia Appendix 1 [file jmir_v27i1e78854_app1.docx]

Search strings:

Instagram

#ecig OR #ecigs OR #e-cig OR #e-cigs OR #ecigarette OR#ecigarettes OR #e-cigarette OR #e-cigarettes OR #vape OR #vapes OR #vaping OR #Vapor OR heatnotburn OR heatedtobacco OR IQOS OR iqos OR #carbonneutral OR #nicotine OR #Nicotine #smokefree OR #tobaccoharmreduction OR #harmreduction OR #THR #vapesaveslives OR #menthol OR #vapelife OR #smokefree OR #smoke-free OR #tobaccofree OR #nicotinefree OR #menthol OR #Menthol OR #mentholban

TikTok

#vape OR#vaping OR#ecig OR#ecigarette OR#e-cig OR#e-cigarette OR#tobacco OR#cig OR#cigarette OR#smoking OR#menthol OR #nicotine

Prompt for Instagram pilot set:

“As a tobacco control expert, please review the content of posts from Instagram. Note that you only need to look at the ‘text’ column, which contains the main claims of a given post. Then use your current knowledge to identify posts that you think contain misinformation. The output should be in a csv file, with URLs to the original post, the text, and a brief explanation of why they are selected.”

Prompts for the 3850 posts:

“As a tobacco control expert, please provide what you would consider as misinformation related to tobacco products.”

“Here are some health guidelines from established organizations regarding tobacco products. Please use these guidelines as references, and also refer to the misinformation selection criteria you provided previously (see below), to review the following dataset that contains Instagram posts about tobacco products, and identify posts that contain misinformation. Please also show the output in a csv file, with URLs to the original post, the text, and a brief explanation of why they are selected.”
